# Reduced brain somatostatin in mood disorders: a common pathophysiological substrate and drug target?

**DOI:** 10.3389/fphar.2013.00110

**Published:** 2013-09-09

**Authors:** Li-Chun Lin, Etienne Sibille

**Affiliations:** Department of Psychiatry, Center for Neuroscience, University of PittsburghPittsburgh, PA, USA

**Keywords:** somatostatin, somatostatin-expressing interneurons, SST, SOM, SRIF, depression, mood disorders, GABA inhibition

## Abstract

Our knowledge of the pathophysiology of affect dysregulation has progressively increased, but the pharmacological treatments remain inadequate. Here, we summarize the current literature on deficits in somatostatin, an inhibitory modulatory neuropeptide, in major depression and other neurological disorders that also include mood disturbances. We focus on direct evidence in the human postmortem brain, and review rodent genetic and pharmacological studies probing the role of the somatostatin system in relation to mood. We also briefly go over pharmacological developments targeting the somatostatin system in peripheral organs and discuss the challenges of targeting the brain somatostatin system. Finally, the fact that somatostatin deficits are frequently observed across neurological disorders suggests a selective cellular vulnerability of somatostatin-expressing neurons. Potential cell intrinsic factors mediating those changes are discussed, including nitric oxide induced oxidative stress, mitochondrial dysfunction, high inflammatory response, high demand for neurotrophic environment, and overall aging processes. Together, based on the co-localization of somatostatin with gamma-aminobutyric acid (GABA), its presence in dendritic-targeting GABA neuron subtypes, and its temporal-specific function, we discuss the possibility that deficits in somatostatin play a central role in cortical local inhibitory circuit deficits leading to abnormal corticolimbic network activity and clinical mood symptoms across neurological disorders.

## INTRODUCTION

**FIGURE 1:**
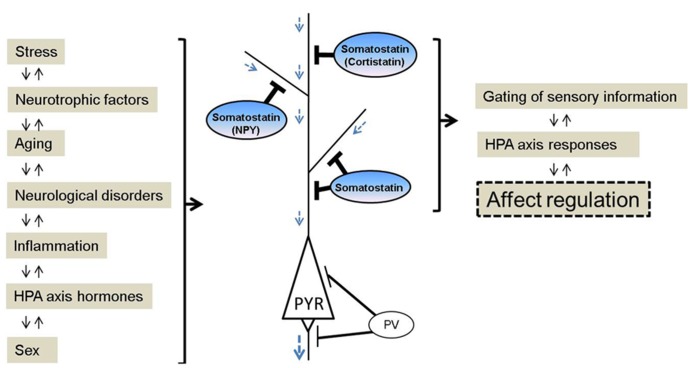
**Schematic of somatostatin signaling, pathological regulators and biological functions relevant to affect regulation.** Somatostatin pathway activity is responsive to (left panel), and regulates (right panel), several biological events, and molecular and cellular properties that have been linked to mood disturbances. Somatostatin and somatostatin-expressing interneurons are key conduits for regulating incoming information and pyramidal cell function. In contrast, other GABA neurons subtypes targeting the perisomatic pyramidal cell compartment are mostly not affected in major depression. NPY, neuropeptide Y; PYR, pyramidal neuron; PV, parvalbumin.

Mood disturbances are commonly observed in many neurological disorders. The chronic, recurrent and long duration of mood disturbances not only place an enormous emotional and financial burden on patients, but also on their families and society. Nearly 10% of all primary care office visits are depression-related (Stafford et al., 2000), but only 30% of patients with mood disturbances achieve remission with initial treatment (Trivedi et al., 2006). Somatostatin is a peptide expressed in multiple organs. In the brain, somatostatin (also known as somatotrophin release inhibiting factor and often abbreviated as SST, SRIF, or SOM) acts as a modulatory and inhibitory neuropeptide that is co-localized with gamma-aminobutyric acid (GABA), and that is involved in regulating multiple aspects of physiological and behavioral stress responses, including inhibition of hypothalamic hormone release, amygdala central nucleus output, and cortical local circuit integration of sensory input. Research advances over the past three decades suggest a critical role for somatostatin in the pathophysiology of mood disorders, and potential new therapeutic strategies. Several recent reviews have summarized the role of the somatostatin system, including in receptor subtypes (Patel, 1999; Csaba and Dournaud, 2001), pharmacological developments (Neggers and van der Lely, 2009), and during normal and pathological aging (Patel, 1999; Viollet et al., 2008; Martel et al., 2012). This article highlights current findings on the functional roles of somatostatin in local neuronal circuits, and reviews somatostatin deficits across neurological disorders, including neuropsychiatric disorders [e.g., major depressive disorder (MDD), bipolar disorder, schizophrenia], and neurodegenerative disorders (e.g., Parkinson’s, Alzheimer’s, and Huntington’s diseases; **Table 1**). This raises interesting questions, including first; whether the somatostatin deficits observed in neurological disorders represent common, distinct, or partly overlapping mechanisms of symptoms across disorders and, second, what may be the causes and biological mechanisms underlying the selective neuronal vulnerability of somatostatin-expressing neurons. In addition, we review somatostatin findings associated with affect regulation at the genetic, cellular, and pharmacological levels in animal studies. So far, these findings suggest that somatostatin deficits across different brain systems and diseases may play a central role in the affective symptom dimension rather than non-specific signals in neurological disorders (**Figure 1**). As somatostatin itself is not an ideal drug target, including for antidepressant effect, we suggest that further studies characterizing the intrinsic properties and biological vulnerabilities of somatostatin-expressing neurons, may identify novel targets with implications for understanding the function of local cell circuits and brain regions underlying affective symptoms across several neurological disorders.

**Table 1:** Low somatostatin in human neurological disorders.

Neurological disorders	Brain region	Pathological findings	Reference
Major depressive disorder	CSF	Decreased	Agren and Lundqvist (1984), Kling et al. (1993), Molchan et al. (1993)
	Dorsolateral prefrontal cortex	Decreased (RNA expression)	Sibille et al. (2011)
	Anterior cingulate cortex	Decreased (RNA expression)	Tripp et al. (2011), Tripp et al. (2012)
	Amygdala	Decreased (RNA and protein expression)	Guilloux et al. (2012)
Schizophrenia	CSF	Decreased	Bissette et al. (1986), Reinikainen et al. (1990)
	Dorsolateral prefrontal cortex	Decreased (RNA expression)	Morris et al. (2008), Guillozet-Bongaarts et al. (2013)
	Hippocampus	Decreased (neuron number and density)	Konradi et al. (2011a)
	Caudal entorhinal cortex	Decreased (neuron number and density)	Wang et al. (2011)
	Parasubiculum	Decreased (neuron number and density)	Wang et al. (2011)
Bipolar disorder	Caudal entorhinal cortex	Decreased (neuron density)	Wang et al. (2011)
	Parasubiculum	Decreased (neuron density)	Wang et al. (2011)
	Hippocampus	Decreased (neuron number and RNA expression)	Konradi et al. (2011b)
	Dorsolateral prefrontal cortex	Decreased (RNA expression; trend level)	Sibille et al. (2011)
Alzheimer’s disease	CSF	Decreased	Bissette et al. (1986); Tamminga et al. (1987)
	Temporal cortex	Decreased (immune-reactivity)	Rossor et al. (1980); Candy et al. (1985)
	Frontal cortex	Decreased (immune-reactivity)	Davies and Terry (1981); Candy et al. (1985)
	Hippocampus	Decreased (gene expression per cell)	Dournaud et al. (1994)
	Parahippocampal cortex	Decreased (neuronal density)	Dournaud et al. (1994)
Parkinson’s disease	CSF	Decreased	Dupont et al. (1982)
	Frontal cortex	Decreased (radioimmune-reactivity)	Epelbaum et al. (1988)
Others	Temporal cortex	Decreased (immune-reactivity)	Beal et al. (1986)

## LOW SOMATOSTATIN IN NEUROPSYCHIATRIC AND NEURODEGENERATIVE DISORDERS

### MAJOR DEPRESSIVE DISORDER

Patients with major depressive disorder (MDD) show decreased somatostatin levels in the cerebrospinal fluid (CSF; Agren and Lundqvist, 1984; Molchan et al., 1991; Kling et al., 1993), and transiently decreased CSF somatostatin which normalize with recovery in MDD (Rubinow et al., 1985; Post et al., 1988). Evidence for low levels of CSF somatostatin was found to correlate significantly with elevated urinary cortisol in MDD patients (Molchan et al., 1993). This is consistent with the altered hypothalamic-pituitary-adrenal (HPA) axis function described in some depressed patients (Holsboer, 2000). The route and characterization, however, from CSF somatostatin to MDD pathophysiology is not direct, potentially due to a paucity of information on factors regulating CSF somatostatin, and to inconclusive somatostatin/HPA axis studies in MDD patients. Hence, despite these early findings, interest in somatostatin in mood disorders has declined over time.

Human post-mortem studies from our group have described region-specific somatostatin deficits in MDD patients, including a down-regulation of *somatostatin *gene expression in the dorsolateral prefrontal cortex (dlPFC), subgenual anterior cingulate cortex (sgACC), and amygdala (Sibille et al., 2011; Tripp et al., 2011, 2012; Guilloux et al., 2012). In addition, two peptides co-localized with somatostatin, neuropeptide Y and cortistatin, are both significantly down-regulated in MDD patients (Tripp et al., 2011, 2012).These three neuropeptides (somatostatin, neuropeptide Y, and cortistatin) are markers of GABAergic neurons that specifically target the dendritic compartment of pyramidal cells (de Lecea et al., 1997; Viollet et al., 2008), and that are essential in gating incoming sensory information (**Figure 1**). Other types of GABAergic cell markers, such as parvalbumin and cholecystokinin, are mostly not affected by MDD (although see Tripp et al., 2012). Interestingly, these somatostatin deficits were systematically more robust in female subjects across cohorts and regions (Sibille et al., 2011; Tripp et al., 2011, 2012; Guilloux et al., 2012), consistent with the female heightened vulnerability to develop MDD, and suggesting that low somatostatin may represent a molecular correlate of sexual dimorphism in vulnerability to affect dysregulation. Notably, these findings are also consistent with earlier postmortem studies showing reduced calbindin-positive cell numbers in MDD (Rajkowska et al., 2007; Maciag et al., 2010), as somatostatin is mostly expressed in a subgroup of calbindin-positive cells (reviewed in Viollet et al., 2008). Converging evidence from down-regulation of *somatostatin *co-localized GABA markers in MDD across multiple human post-mortem studies suggests that this particular GABA subpopulation in the forebrain is selectively vulnerable, among other subtypes of GABA neurons. Furthermore, these local cell circuit-based findings introduce a new role for somatostatin in depression, which is distinct from its previously investigated role in the regulation of the HPA axis (Rubinow et al., 1983; Molchan et al., 1993; Weckbecker et al. 2003).

### OTHER NEUROPSYCHIATRIC DISORDERS

Schizophrenia is a neuropsychiatric disorder characterized by positive (e.g., hallucination), negative symptoms (e.g., emotional blunting, apathy) and cognitive symptoms. Somatostatin deficits in schizophrenia are demonstrated by a reduction of CSF somatostatin (Bissette et al., 1986; Reinikainen et al., 1990), decreased *somatostatin* gene expression in the dlPFC (Morris et al., 2008; Guillozet-Bongaarts et al., 2013), and decreased number and density of somatostatin-expressing neurons in the hippocampus (Konradi et al., 2011a), caudal entorhinal cortex and parasubiculum (Wang et al., 2011). Changes in somatostatin are also identified in bipolar disorder, which is clinically characterized by fluctuating mood. Studies in subjects with bipolar disorder indicate decreases in somatostatin cellular density in the caudal entorhinal cortex and parasubiculum (Wang et al., 2011), number of somatostatin-expressing neurons in the hippocampus (Konradi et al., 2011b), *somatostatin* gene expression in the dlPFC (trend level; Sibille et al., 2011) and hippocampus (Konradi et al., 2011b). In addition, patients with bipolar disorder show elevated CSF somatostatin during manic states (Sharma et al., 1995).

### NEURODEGENERATIVE DISORDERS

Alzheimer’s disease is a neurodegenerative disease with neuropsychiatric symptoms (Bungener et al., 1996). Decreased CSF somatostatin (Bissette et al., 1986; Tamminga et al., 1987) and decreased somatostatin immune-reactivity across cortical and subcortical regions is reported in subjects with Alzheimer’s disease, including temporal cortex, frontal cortex, and hippocampus (Davies et al., 1980; Rossor et al., 1980; Davies and Terry, 1981; Candy et al., 1985; Dournaud et al., 1994). Depression is a common comorbid symptom in Parkinson’s disease and predicts greater disability at any assessment point (Aarsland et al., 1999). Decreased CSF somatostatin, decreased somatostatin immuno-reactivity, and binding sites are also observed in the temporal cortex and frontal cortex of patients with Parkinson’s disease (Beal et al., 1986; Epelbaum et al., 1988). Notably, reduced CSF somatostatin in Parkinson’s disease appears to be irreversibly present at the onset of symptoms (Dupont et al., 1982).

### REDUCED SOMATOSTATIN AND LOW MOOD?

The evidence outlined in this review provides only a glimpse of the potential full range of somatostatin deficits across neurological disorders, as multiple other brain regions and disease categories await further characterization (**Table 1**). Taken together, the cumulative evidence demonstrates that somatostatin deficits are common neurochemical and molecular features in individuals with neurological disorders, regardless of their categorical diagnosis. While somatostatin studies of cell number and gene expression in human postmortem brains suggest a specific alteration of somatostatin-positive neurons across neurological disorders, it is possible that changes and dys-synchronization of additional components of local neuronal circuits contribute to a common symptom dimension, which we speculate includes low affect and mood dysregulation. Hence, this review is not comprehensive, but rather, highlights the recent findings in brain somatostatin signaling and the potential role of somatostatin deficits in affect dysregulation for integrating categorical models of mood symptoms into a dimensional model across neurological disorders.

## SOMATOSTATIN: GENES, NEURONS AND PHARMACOLOGY

### SOMATOSTATIN SIGNALING

Somatostatin is a modulatory neuropeptide that synergizes with GABA-mediated inhibition, and that specifically targets the distal dendritic compartment of pyramidal neurons in cortical local circuits (Kawaguchi and Kubota, 1997; Gentet et al., 2012). Somatostatin inhibits release of numerous hormones from the hypothalamus, including corticotrophin releasing hormone (CRH; Wang et al., 1987; Patel, 1999). The somatostatin gene product is composed of 14 or 28 amino-acid residues. Both forms of somatostatin, somatostatin-14 and somatostatin-28, are generated by tissue-specific post-translational processing of the 116 amino-acid pre-pro-somatostatin peptide (Warren and Shields, 1984; Tostivint et al., 2008). Somatostatin-14 is predominantly produced in the central nervous system (CNS) but also in many peripheral organs (Epelbaum, 1986). Somatostatin-28 is mainly synthesized along the gastrointestinal tract (Fitz-Patrick and Patel, 1981). The 5′-upstream sequence of the *somatostatin *gene contains cyclic-AMP response element (CRE; Montminy et al., 1986), making its expression activity-dependent. Thus, *somatostatin *expression is preferentially altered by various stressors, such as seizures (Vezzani and Hoyer, 1999; Tallent and Qiu, 2008) and electrical foot shock (Ponomarev et al., 2010). Moreover, mice with conditional homozygous and constitutive heterozygous brain-derived neurotrophic factor (*Bdnf*) knockout or disruption of exon IV-expressing *Bdnf *transcripts show decreased *somatostatin* gene expression (Glorioso et al., 2006; Martinowich et al., 2011; Guilloux et al., 2012), demonstrating that *somatostatin* expression depends on *Bdnf *signaling. However, the molecular mechanisms by which this neurotrophic factor controls somatostatin and somatostatin-expressing neurons are still unknown.

Somatostatin, cortistatin and their receptors are closely intertwined systems (de Lecea et al., 1996, 1997; reviewed in Spier and de Lecea, 2000; de Lecea, 2008). Sharing high structural homology with somatostatin, cortistatin binds to all somatostatin receptor subtypes and is known to be regulated by exon IV-expressing *Bdnf *transcripts (Martinowich et al., 2011). However, distinct from somatostatin, cortistatin binds to additional receptors (e.g., growth hormone secretagogue receptor 1a and Mas-related gene X2 receptor) (Robas et al., 2003; Siehler et al., 2008) and has different physiological properties (e.g., activation of cation selective currents not responsive to somatostatin; Spier and de Lecea, 2000), suggesting that somatostatin and cortistatin may both contribute to affect regulation in an integrated, yet differential mode. The intracellular pathway of somatostatin signaling coupled to all five somatostatin receptors subtypes (Sst_1__-__5_) is through the activation of inhibitory G protein (Gi) and the following inhibition of adenylyl cyclase, leading to reduction of cAMP levels, activation of phosphotyrosine phosphatases, and modulation of mitogen-activated protein kinases and phospholipase C (Koch and Schonbrunn, 1984; Koch et al., 1988).

Sst_1-5_ present different patterns of coexpression in the brain (Kluxen et al., 1992; Moller et al., 2003; reviewed in Martel et al., 2012). Sst1 is found in retina, basal ganglia and hypothalamus, Sst2 is highly abundant in several telencephalic structures (neocortex, hippocampus, and amygdala), Sst3 immunoreactivity has only been described in neuronal cilia (Schulz et al., 2000), Sst4 is expressed in olfactory bulb, cerebral cortex and CA1 region of the hippocampus (Schreff et al., 2000), and expression of Sst5 has been detected in cerebral cortex, hippocampus, amygdala, preoptic area, and hypothalamus (Stroh et al., 1999; Strowski et al., 2003; Olias et al., 2004). Interestingly, when co-expressed in the same cells, Sst5 influences Sst2 internalization and trafficking and modulates cellular desensitization to the effects of somatostatin-14 (Sharif et al., 2007), suggesting that the precise actions of somatostatin depend on the specific interaction of the Sst_1-5_ receptors expressed locally in each brain region.

### GENETIC POLYMORPHISMS IN THE SOMATOSTATIN SYSTEM

The relatively high degree of amino acid conservation across species indicates that somatostatin-related genes have been highly constrained during evolution (Patel, 1999; Olias et al., 2004). Accordingly, there are currently very few reports linking *somatostatin *gene polymorphisms with neurological disorders. A primate-specific single nucleotide polymorphism (SNP) in the human *somatostatin* gene [C/T polymorphism (rs4988514)] is associated with increased risk in Alzheimer’s disease progression and additive effect with the APOE epsilon4 allele(Vepsalainen et al., 2007; Xue et al., 2009), although this was not confirmed in larger genome-wide association studies (GWAS) (Hollingworth et al., 2011; Guerreiro et al., 2013). Leu48Met and Pro335Leu SNPs in the *SST5 *gene are of potential significance to patients with bipolar disorder (Nyegaard et al., 2002), but no associations of SST5 SNPs are found in patients with autism (Lauritsen et al., 2003). The paucity of associations with somatostatin gene variants is surprising and may reflect either strong negative selection against genetic variations in this gene, or alternatively, dilution of signal due to heterogeneity of DSM-IV-based cohorts in genetic association studies. So, dimensional phenotypes, as defined by clusters of mood symptoms, which are closer to gene functions may have implications for future genetic studies of somatostatin and other genes.

### SOMATOSTATIN-EXPRESSING NEURONS: DIVERSITY AND ROLES

Gamma-aminobutyric acid (GABA) neurons are a diverse group of inhibitory cells which co-release neuropeptides in order to support a fine-tuning of neuronal signaling and architecture. The local inhibitory circuits provide spatiotemporal control of information processing through at least 20 subtypes of cortical GABA neurons, which are based on their expression of different calcium binding proteins and neuropeptides, localization, targeting, and differential electrophysiological properties. Recent detailed reviews on GABA neuron subpopulations have been published (Csaba and Dournaud, 2001; Di Cristo et al., 2004; Markram et al., 2004; Tan et al., 2008; Fishell and Rudy, 2011; Gentet et al., 2012; DeFelipe et al., 2013; Le Magueresse and Monyer, 2013). Approximately 20–30% of GABA neurons in the mouse somatosensory cortex express somatostatin (Lee et al., 2010; Rudy et al., 2011), and 40–50% of GABA neurons contain parvalbumin without overlapping with somatostatin in the frontal cortex, primary somatosensory cortex and visual cortex of mouse (Gonchar et al., 2007; Xu et al., 2010) and the visual cortex of rat (Gonchar and Burkhalter, 1997).

Recent reports focusing on the patterns of cortical neuronal connectivity show that somatostatin-expressing interneurons mediate the firing of pyramidal neurons with a fine level of specificity among cortical layers. Integrating optogenetic and electrophysiology approaches, mouse somatostatin-expressing interneurons in layer 2/3 of the somatosensory cortex provide a tonic inhibition to the distal dendrites of excitatory pyramidal neurons by sharpening selectivity during periods of quiet wakefulness, which may contribute to synchronized firing in cortical networks and sensorimotor integration (Gentet et al., 2012). Interestingly, in mouse somatosensory cortex, somatostatin-expressing interneurons show a spatially precise connectivity with pyramidal neurons through direct targeting in layers 2/3 or indirectly through inhibition of local parvalbumin interneurons in layer 4 (Xu et al., 2013). Moreover, in layers 2/3 of the mouse prefrontal cortex, somatostatin-expressing interneurons compartmentalizes inhibitions of calcium signaling to spine heads, not shafts, suggesting that dendrite-targeting inhibition through somatostatin-expressing interneurons may contribute to downstream cellular processes such as synaptic plasticity (Chiu et al., 2013). In mouse visual cortex, somatostatin-expressing interneurons are found to mediate response levels of specific subsets of pyramidal neurons whereas parvalbumin-expressing neurons alter response gain (Wilson et al., 2012). Parvalbumin-expressing neurons receive excitatory input from the thalamus and make strong synapses on the soma and axons of their target cells (Kawaguchi and Kubota, 1997) to control spike timing of the output neurons. In contrast, somatostatin-expressing neurons mostly do not receive input from thalamus (Beierlein et al., 2003; Cruikshank et al., 2010) and are instead activated through feed-forward mechanisms by activated pyramidal neurons. Somatostatin-expressing interneurons preferentially target distal dendrites of pyramidal neurons in layer 2/3 to modulate the processing of incoming sensory information before it is integrated at the soma level (Di Cristo et al., 2004; Markram et al., 2004; Tan et al., 2008; Murayama et al., 2009; Xu et al., 2013). Hence, the distinct GABAergic and prototypical inhibitory populations, expressing either parvalbumin or somatostatin, shape the spatiotemporal control of multiple post-synaptic potentials in cortical local circuits, and provide a framework to investigate the role of inhibitory circuits in physiology and pathology.

### GENETIC APPROACHES TO INVESTIGATE THE SOMATOSTATIN SYSTEM

Mice mutant for somatostatin were created by deleting the coding region of the pre-pro-somatostatin (the last ten codons of the first exon; Zeyda et al., 2001). Somatostatin knockout (KO; Sst^KO^) mice show intact motor coordination and motor learning, but have a significant impairment in motor learning as demands of motor coordination are increased. Overall, a detailed analysis demonstrated that Sst^KO^ mice are healthy, fertile, and show no overt behavioral phenotypes, including anxiety-like behavior in the open-field and fear conditioning tests. Notably, Sst^KO^ mice display high basal plasma levels of corticosterone and growth hormone (Zeyda et al., 2001), confirming a somatostatin-mediated inhibition of HPA axis function. Similarly, mice lacking individual Sst_1__-__5_ receptors have been tested in numerous biological fields. Of these, Sst_2_ emerged as the primary receptor of interest (Zeyda and Hochgeschwender, 2008), and Sst_2_^KO^ mice display increased anxiety-like behavior in the elevated plus maze and open field, increased immobility in the forced swim test, decreased locomotion coupled with an increase of pituitary adrenocorticotropic hormone release instead of growth hormone (Viollet et al., 2000). In line with the observed changes in Sst_2_^KO^ mice, acute predator stress in rats led to up-regulated *Sst*_2_ gene expression in the amygdala and cingulate cortex, shown correlated with Fos expression in the amygdala (Nanda et al., 2008). As the product of a different gene, cortistatin shares a high structural and functional similarity with somatostatin-14 (de Lecea et al., 1996, 1997). Notably, compared with the weak inhibitory effects of somatostatin on the basal release of CRH from rat hypothalamus and hippocampus, cortistatin exhibits strong inhibition of the expression and release of basal CRH (Tringali et al., 2012). These findings suggest that Sst2 may regulate affective phenotypes and HPA axis responses both through somatostatin and cortistatin. Given the limitations of human studies, Sst^KO^ mice provide an opportunity to explore the causal role of somatostatin in affect dysregulation and the underlying neural mechanisms. Such insights, however, will require systematic behavioral characterization with fine spatial and temporal resolution by including female cohorts and region-specific manipulation at different developmental stages. Based on the published studies to date, it is still unclear whether these mutants recapitulate behavioral features of mood disorders. Knowing the effects of somatostatin signaling on neuroendocrine regulation, future studies need to assess the molecular and cellular systems that somatostatin mutations converge upon, and where the exact neural circuits are affected. Moreover, combining genetic and environmental factors in animal models is critical to enhance the accuracy of disease modeling and translational efforts. For example, acute or chronic exposure to stress or to stress hormones may capture how such etiological factors determine the vulnerability to external insults, in contrast to baseline behavioral testing. In addition, mood disorder-related sex differences are observed in community-based epidemiological studies, where the factor of seeking treatment is removed (Kornstein et al., 2000; Festinger et al., 2008; Leach et al., 2008) and findings of low somatostatin in the amygdala appear more robust in postmortem studies of female MDD subjects (Tripp et al., 2012), suggesting that gender/sex may represent a biological predisposing factor, or at least a moderating factor, in the intrinsic vulnerability of the somatostatin system.

Although many mood disorders emerge during adolescence (Paus et al., 2008), behavioral abnormalities including affect dysregulation are often heritable and apparent before diagnostic criteria are met (McGuffin et al., 2003; Geller et al., 2006). It is unclear when somatostatin deficits occur and potentially begin to contribute to the formation of affective symptoms. Tracking somatostatin system using new anatomic techniques with refined cellular definition, from Brainbow (Livet et al., 2007) to CLARITY (Chung et al., 2013) and SeeDB (Ke et al., 2013), across different developmental stages may help identify age-dependent neural architecture and disease mechanisms related to somatostatin function.

### SOMATOSTATIN ANALOG DEVELOPMENT AND PHARMACOLOGICAL STUDIES

As native somatostatin peptides have a very short half-life time (approximate 1–3 min; Sheppard et al., 1979), long-acting and highly potent somatostatin analogues are currently available for the treatment of acromegaly and neuroendocrine tumors, including octreotide (long-acting; LAR-OCT; Bauer et al., 1982) and Lanreotide (slow release or autogel; Bevan, 2005; Molitch, 2008). Compared to somatostatin, pharmacological tools of the five somatostatin receptor subtypes have lagged behind, partly due to the lack of high-affinity antagonists.

In addition, several novel somatostatin therapy models are available: (1) Universal somatostatin (Schmid and Schoeffter, 2004): a somatostatin molecular analog with high binding affinity to all or most human somatostatin receptors. An example is SOM230, which interacts with Sst_1,2,3,5_ and particularly potent at Sst_5_ compared with LAR-OCT; (2) Chimeric somatostatin/dopamine molecule (Saveanu et al., 2002; Pivonello et al., 2005): a somatostatin and dopamine hybrid agonist, based on reports that dopamine and somatostatin receptors can hetero-oligomerize to enhance functional responses (Rocheville et al., 2000). An example is BIM-23A760, which accelerates the suppression of growth hormone and adrenocorticotropic hormone by the interaction with Sst_2_ and Drd2 simultaneously; (3) Chimeric-somatostatin vaccinations (Haffer, 2012): a fusion protein expressing chloramphenicol acetyl transferase protein and somatostatin. Two somatostatin vaccinations, JH17 and JH18, can effectively reduce weight gain and reduce final body weight percentage of normal, non-obese mice and mice with diet-induced obesity via the intra-peritoneal route; (4) Non-peptide antagonists, such as SRA880 (Sst_1_ selective), ACQ090 (Sst_3_ selective) and Sst_4_ selective β peptide agonists (Rivier et al., 2003; Hoyer et al., 2004). Despite this extensive list, the practical use of somatostatin in the brain is hampered by the multiple effects of the peptide, by the need for small molecules targeting specific, high affinity receptors on the target cells in specific brain regions, and by the need for feasible routes of administration that lead to fast delivery into the brain.

The potential for using somatostatin analogues as treatment in the CNS is emerging for treatment of epilepsy (Vezzani and Hoyer, 1999; Tallent and Qiu, 2008), pain (Mollenholt et al., 1994; Taura et al., 1994) and headaches (Sicuteri et al., 1984; Kapicioglu et al., 1997); potential use for treatment of mood disorders is suggested by reversal of emotion-like behaviors in rodent models. Several pharmacological studies support a role of somatostatin in affect regulation. Intra-ventricular administration of somatostatin in rats produces anxiolytic- and antidepressant-like behaviors in the elevated plus-maze and forced swim tests, and a neurophysiological signature of anxiolytic drugs (e.g., reduction of theta frequency and theta frequency curve slope; Engin et al., 2008). Mice with intra-amygdalar and intra-septal microinfusions of somatostatin-14 and somatostatin-28 display reduced anxiety-like behavior in the elevated plus-maze and shock-probe tests (Yeung et al., 2011). Moreover, anxiolytic effects in the elevated plus-maze test are described after intra-cerebroventricular infusions of a selective Sst_2_ receptor agonist, but not after infusions of the other four receptor agonists; antidepressant-like effects in the forced swim test are observed following infusions of either Sst_2_ or Sst_3_ agonists (Engin and Treit, 2009). Another agent to enhance somatostatin functioning, SRA880 (an antagonist of auto-receptor Sst_1_), synergizes with imipramine in causing antidepressant-like effects in the tail suspension test and increases *Bdnf* mRNA expression in the mouse cerebral cortex (Nilsson et al., 2012).

### EFFECTS OF ANTIDEPRESSANTS ON SOMATOSTATIN IN THE CNS

Significant efforts have been directed toward the characterization of the downstream targets of antidepressant treatment, with a focus on somatostatin. A recent study demonstrates that chronic imipramine treatment increases somatostatin expression in mouse hypothalamus (Nilsson et al., 2012). However, there is inconsistency regarding the effect of chronic citalopram treatment on somatostatin levels in rats (Kakigi et al., 1992; Prosperini et al., 1997; Pallis et al., 2006, 2009). Repeated administration of imipramine, maprotiline, mianserin, carbamazepine or zotepine has no effect on somatostatin levels in various brain regions of rats (Weiss et al., 1987; Kakigi et al., 1992). While some somatostatin receptors seem to exert anxiolytic or antidepressant-like effects, there is no direct evidence supporting somatostatin receptors as downstream targets of current antidepressants. Together, these findings suggest that somatostatin levels are mostly unchanged by antidepressants. It is unclear whether somatostatin, GABA, or GABA functioning in somatostatin-expressing interneurons may be the real mediators or antidepressant targets. Future studies are needed to determine the involvement of somatostatin receptors and associated intracellular signaling pathways in the therapeutic effects of antidepressants, or whether somatostatin effects are independent of current antidepressant modalities.

## POTENTIAL MECHANISMS OF SELECTIVE VULNERABILITY OF SOMATOSTATIN-EXPRESSING INTERNEURONS

It is possible that low somatostatin in diseases acts as a biomarker for deregulated function of somatostatin-expressing neurons. As such, it is essential to identify upstream factors responsible for the dysfunction of somatostatin-expressing interneurons in neurological disorders. We speculate that intrinsic cellular properties in somatostatin-expressing neurons may determine their selective vulnerability to various insults. Pathways underlying this high vulnerability may include high intrinsic oxidative stress related to mitochondria, high sensitivity to inflammation, high dependence on neurotrophic environment, and cellular developmental and aging processes. These canonical pathways might provide novel cell-based perspectives in the treatment of affected somatostatin-expressing cells across neurological disorders.

### OXIDATIVE STRESS AND MITOCHONDRIAL DYSFUNCTIONS

Oxidative stress produced by mitochondria during respiration is a common pathogenic mechanism implicated in neurological disorders (Sorce and Krause, 2009; Stefanescu and Ciobica, 2012). Depressed states in mood disorders are associated with decreased brain energy generation (Baxter et al., 1985, 1989). Mitochondrial dysfunction together with the oxidative stress accumulation has been proposed to synergistically contribute to the neuro-endangerment processes underlying depression (Gardner et al., 2003; Burnett et al., 2005) and neurodegenerative diseases (Lin and Beal, 2006; Mancuso et al., 2007; Petrozzi et al., 2007). Similarly, high baseline oxidative stress could be an intrinsic characteristic of vulnerable neuronal populations. Notably, neuronal nitric oxide synthase (nNOS) and NADPH diaphorase (NADPHd), two enzymes that produce reactive oxidative species, are extensively and almost exclusively co-localized with somatostatin and neuropeptide Y (Dun et al., 1994; Figueredo-Cardenas et al., 1996; Jaglin et al., 2012), hence providing a neurochemical basis for high susceptibility of somatostatin-expressing neurons to generate oxidative stress in response to pathophysiological insults.

### HIGH DEPENDENCE ON NEUROTROPHIC ENVIRONMENT

Brain-derived neurotrophic factor (BDNF) and its receptor neurotrophic tyrosine kinase receptor type 2 (TrkB) have been implicated in mood disorders (Guilloux et al., 2012; Tripp et al., 2012). BDNF-TrkB signaling is one of the key mediators for maintaining normal *somatostatin* gene expression (Glorioso et al., 2006; Martinowich et al., 2011). Progressively impairing BDNF-TrkB signaling in patients with mood disturbances may directly impact the biology of somatostatin-expressing neurons, resulting in somatostatin deficits. In addition, Bdnf-TrkB signaling itself is vulnerable to increased inflammation (Goshen et al., 2008; Koo and Duman, 2008; Song and Wang, 2011) and high glucocorticoids insults (Hodes et al., 2012). Mild oxidative stress inhibits tyrosine phosphatases activity (Barrett et al., 2005), potentially leading to impaired TrkB downstream signaling. Cortistatin and neuropeptide Y expression partly overlaps with the somatostatin neuron population in rodents (Figueredo-Cardenas et al., 1996; de Lecea et al., 1997; Xu et al., 2010). Comparing the profile of gene changes between subjects with MDD and mice with genetically-altered Bdnf signaling suggest that the reduced *somatostatin*, *neuropeptide Y* and *cortistatin* are partly downstream from a combination of reduced constitutive and activity-dependent Bdnf signaling (Guilloux et al., 2012). In contrast, markers for other GABA neuron subtypes targeting the perisomatic area region cell body and axon initial segment of pyramidal neurons (i.e., cholecystokinin and calretinin), appear to be independent of BDNF signaling and unaffected in MDD patients (Guilloux et al., 2012; Tripp et al., 2012). Hence, it is possible that the somatostatin-specific cellular function and vulnerability are partly mediated by BDNF-TrkB signaling during both physiological and pathological processes of affect regulation.

### INFLAMMATION AND CELLULAR AGING

Inflammation has been implicated as a contributing factor in the onset and progression of many neurological disorders (Di Filippo et al., 2008). Mood disturbances are associated with an activated inflammatory response system (Padmos et al., 2008; Miller et al., 2009), including increased levels of peripheral interleukins and tumor necrosis factor-alpha in MDD patients (Kaestner et al., 2005; Howren et al., 2009; Dowlati et al., 2010; Maes, 2011). Inflammatory illnesses are associated with more depressive episodes (Celik et al., 2010; Maes et al., 2012), suggesting that prior depression may sensitize inflammatory responses. Patients treated with inflammatory cytokines, such as interferon-α, are at greater risk of developing depressive episodes (Castera et al., 2006; Lotrich et al., 2007). Somatostatin released from sensory nerves and somatostatin receptors on peripheral blood mononuclear cells play a crucial role in anti-inflammation through inhibition of pro-inflammatory peptide release (Szolcsanyi et al., 1998; Kurnatowska and Pawlikowski, 2000; Helyes et al., 2004). Rats with chronic inflammation induced by lipopolysaccharide show decreased hippocampal *somatostatin* expression (Gavilan et al., 2007). It is possible that there is crosstalk among peripheral inflammation, somatostatin function, and central effects of somatostatin-expressing neurons. Hence, decreasing somatostatin expression due to cellular impairment in the progress of neurological diseases may further enhance inflammation in a vicious cycle, leading to exacerbated cellular vulnerability of somatostatin-expressing neurons.

Aging is associated with a considerable increase in an activated, pro-inflammatory state (Wei et al., 1992; Bruunsgaard and Pedersen, 2003), a decline in circulating levels of Bdnf (Erickson et al., 2010), and increased oxidative damage (Sohal and Weindruch, 1996). *Somatostatin* expression is significantly decreased with age in human cortical regions, but parvalbumin expression is not altered by age (Erraji-Benchekroun et al., 2005; Glorioso et al., 2011). Similarly, the number of hippocampal somatostatin-expressing interneurons decreases in aged rats, but the number of parvalbumin-expressing neurons remains the same (Vela et al., 2003). *Somatostatin* and *IL-1*β mRNA expression are negatively correlated in aged hippocampus of rats (Gavilan et al., 2007). Comparing the effects of aging on *somatostatin *expression in the sgACC, an accelerated reduction is found in patients with MDD compared to normal aging subjects (Tripp et al., 2012), suggesting a pattern resulting in an early aging phenomenon which we have speculated may be synergistically induced by normal age-related changes and depression-related pathological change (Douillard-Guilloux et al., 2013).

## CONCLUSION

Here we have focused on somatostatin, a GABA marker, down-regulated in MDD, schizophrenia, bipolar disorder, and neurodegenerative diseases. Exploring cross-disease molecular (somatostatin) and cellular (somatostatin-expressing interneurons) pathological findings suggests a dimensional pathological phenotype that is specific to the somatostatin gene/cell biological entity rather than to categorical brain disorders. Based on these results we speculate that common risk factors affecting somatostatin and somatostatin-expressing neurons may impact information processing in the cortical local circuits (**Figure 1**). Clarifying the role of somatostatin and its regulation of GABA inhibition in affect regulation could provide new strategies for predicting, delaying, and treating neurological diseases with mood disturbances. A number of questions remain. For example, are the prevalent somatostatin deficits seen in multiple diseases reflected in a common symptom dimension, such as low mood, across neurological diseases? What are the critical events that determine the vulnerability of somatostatin-expressing neurons? And what are the pathogenic mechanisms that mediate the observed disease-related molecular and cellular phenotypes? One possibility is that inflammation, oxidative stress, aging, and reduced neurotrophic support may all converge to affect somatostatin-expressing neurons. Targeting these pathways may exert neuro-protective effects on somatostatin-expressing neurons, as a potential therapeutic approach with implications for several neuropsychiatric disorders and neurodegenerative diseases.

## Conflict of Interest Statement

The authors declare that the research was conducted in the absence of any commercial or financial relationships that could be construed as a potential conflict of interest.
